# Clinical Presentations and Outcomes of Coronavirus Disease 2019 in Patients With Solid Tumors

**DOI:** 10.7759/cureus.15452

**Published:** 2021-06-05

**Authors:** Imran Farooque, Umar Farooque, Sundas Karimi, Muhammad Usman Shah Syed, Zubia Nadeem, Arif Zulfiqar, Sufyan Mustafa, Rizwan Farooque, Ayyaz A Sultan, Syed Adeel Hassan

**Affiliations:** 1 Public Health, Peoples University of Medical and Health Sciences for Women, Nawabshah, PAK; 2 Neurology, Dow University of Health Sciences, Karachi, PAK; 3 Orthopedic Surgery, Dow University Hospital, Karachi, PAK; 4 Internal Medicine, Dow University of Health Sciences, Karachi, PAK; 5 Medicine, Dow University of Health Sciences, Karachi, PAK; 6 Medicine and Surgery, Dow Medical College, Karachi, PAK; 7 Medicine, Dow Medical College, Civil Hospital, Karachi, PAK; 8 Internal Medicine, Sindh Medical College, Karachi, PAK; 9 Hematology/Oncology, California Cancer Associates for Research & Excellence, Fresno, USA; 10 Internal Medicine, University of Louisville, Louisville, USA

**Keywords:** clinical presentation, outcomes, mortality, pandemic, covid-19, solid tumor, prostate cancer, colorectal cancer, breast cancer, lung cancer

## Abstract

Background

Coronavirus disease 2019 (COVID-19) is a global health crisis. The literature suggests that cancer patients are more prone to be affected by COVID-19 because cancer suppresses the immune system and such patients usually present poor results. The objective of this study is to present all clinical, laboratory, and demographic characteristics of COVID-19 patients with solid tumors.

Methodology

This study was conducted at the Dow University of Health Sciences for a period of six months from April 2020 to September 2020. In this study, we included a total of 1,519 confirmed patients diagnosed with solid tumors via polymerase chain reaction. The mortality timeline within 30 days of contracting the virus was considered, and the median age of the included individuals was 61 years, with a range of 20-95 years. Of the patients included in the study, 49.4% (750) were men; moreover, 3.15% of our study population had prostate cancer, 10.20% had colorectal cancer, 2.76% had breast cancer, and 10.46% had lung cancer. Of the patients, 25.93% presented with at least one comorbidity. For 73% of the patients, at least one direct therapy for COVID-19 was included in the treatment; 56.6% of the patients were hospitalized, and 11.32% were admitted to the intensive care unit.

Results

The mortality rate was 4.74% in the first 30 days after diagnosis, where 72 patients died. The findings of the first multi-variation model showed that males at older ages who were diabetic and going through cytotoxic therapy were prone to die within the first 30 days. However, the 30-day mortality rate was lower in patients diagnosed with prostate and breast cancer. The second set incorporated laboratory factors, where we found that higher values of leukocytosis, thrombocytopenia, and lymphocytopenia were correlated with higher rates of mortality within 30 days.

Conclusions

We conclude that there is a higher mortality rate of COVID-19 in patients with solid tumors than in the general population. However, it was found to be lower in the Pakistani population compared with the Chinese and Western populations. Intensive care can decrease mortality rates in COVID-19 and cancer patients.

## Introduction

Coronavirus disease 2019 (COVID-19) is a recent pandemic that has turned out to be a global health crisis. It is a severe acute respiratory syndrome that was reported for the first time in Wuhan, China, in December 2019 and shortly after spread around the globe [1]. Most reports of COVID-19 suggest that this virus affects patients differently according to age and comorbidities. Specifically, it affects people who are older and have comorbidities that are associated with higher risks of mortality and infection. Cancer is a high-risk disease in which patients are at a greater risk because of such vulnerabilities as immunodeficiency. Cancer suppresses patients’ immune systems, especially when they are older and have frequent diseases. Early reports on COVID-19 suggest that cancer patients are prone to be affected by COVID-19, and such patients usually present poor results [2-8]. As COVID-19 spreads rapidly and has a higher prevalence in cancer patients around the globe, it is imperative to define the risk factors, outcomes, and features of this group. To accomplish this, in Pakistan, the Ministry of Health formulated a registry of patients nationwide who had COVID-19 in the earliest days of the pandemic. All cases of COVID-19 confirmed by positive polymerase chain reaction (PCR) were registered and managed by the physicians who attended them. The data used in our study, including clinical, demographic, and laboratory data of patients who presented with solid malignancy and positive COVID-19 confirmed by PCR, were collected from the national registry of COVID-19 patients in Pakistan.

## Materials and methods

On February 26, 2020, the first case of COVID-19 was reported in Pakistan, and the Ministry of Health responded swiftly by devising a national COVID-19 registry. This was established to develop a database to monitor cases, testing, and strategies to devise a treatment for the disease. The prospective registration of COVID-19 cases in all private and government hospitals was conducted, and cases that had been previously diagnosed were added to the registry. This form-based registry is ongoing, and attending clinicians are updating the database electronically on a periodic basis.

The data collected after identification have been made available to investigators for further analysis. All the patients included in this data were accurately diagnosed with confirmed PCR analysis. The samples were obtained via nasal swabs. We did not include patients who presented with negative PCR results, but they were recorded in the registry.

The timeline of this study was between April and September 2020. It was conducted at the Dow University of Health Sciences after ethical approval from the Institutional Review Board, and the informed consent requirement was waived. The study included adult patients (≥18 years) with solid tumors who were confirmed COVID-19 cases. For analysis of solid tumors, codes from the International Classification of Diseases, 10th edition were used. Patients who presented with in situ carcinomas, as well as those with malignancies related to hematology, were excluded from the study.

We assessed features including demographic treatment information for cancer and COVID-19. The assessment of laboratory tests included coagulation tests, CT scans, biochemistry serum tests, and blood counts. The reference values of the corresponding centers were used to categorize laboratory results to consider normal and abnormal reference values. The CT scans were classified as “normal,” “consistent with viral pneumonia,” or “other findings.” We did not use a predefined criterion to classify CT scans; it was solely at the discretion of the local hospital radiologist. There was no CT scan central review. To extract the information on cancer treatment, Anatomical Therapeutic Chemical codes were used from the existing database. We defined “receiving active cancer treatment” as patients being treated for cancer within four weeks of diagnosis of COVID-19. Details of COVID-19 treatment, including azithromycin, remdesivir, and hydroxychloroquine (HCQ), and information regarding intensive care unit (ICU) admission were collected. The follow-up period was defined from the first confirmed PCR test date until 30 days after this test. The patients included in the study were those who were followed up for at least 30 days and those who died within 30 days of continuing COVID-19. The final outcome of the study was 30-day mortality.

Statistical analysis

The mean ± standard deviation (SD) was used to characterize the baseline clinical and demographic variables and frequencies for categorization. To determine comorbidities, sex, age, lab parameters, and the treatment methods correlated with 30-day mortality, univariate analysis, chi-square test, and Student’s t-test were used. To determine the relevant clinical factors correlated with mortality within 30 days, multivariate analysis using logistic regression was used. Certain variables that showed significant correlation levels of α = 0.020 were included in the multivariate model. Data completeness and clinical validation were also included. A backward selection was used in the logistic regression analysis in the multivariate model. To assess the model fit, Hosmer−Lemeshow goodness-of-fit statistics were used. For statistically significant influence, the type 1 error level, which was less than 5%, was used. The Statistical Package for the Social Sciences version 24 (IBM Corp., Armonk, NY) was employed to perform all statistical analyses.

## Results

The total number of participants was 1,519. The age, gender, demography, and clinical and treatment characteristics of the patients are shown in Table 1. The participants’ ages were in the range of 20-95 years, and the median age was 62 years. Patients older than 65 years made up 38.8% of the sample, and 750 were men. Of the patients in our study population, 3.15% had prostate cancer, 10.20% had colorectal cancer, 2.76% had breast cancer, and 10.46% had lung cancer. Moreover, 25.93% of the patients presented with at least one comorbidity. The most common comorbid disease was hypertension at 25.7%, followed by diabetes mellitus (12.7%) and chronic obstructive pulmonary disease (6.84%). The least common was kidney disease at 4.73%. Clinical features correlated with high mortality risk were hypertension, chronic liver disease, diabetes, and chronic kidney disease, which were found in older men. The university centers’ mortality rate was 9.7%, and the public hospital mortality rate was 3.9% (p = 0.002). Overall, 25% of the patients had received systemic anti-cancer therapy within four weeks before the diagnosis of COVID-19; in this context, 13.5% received cytotoxic therapy, 11.1% received hormone therapy, and the remaining 3.6% received targeted therapy. Immunotherapy was administered to only two patients.

**Table 1:** Analysis of age, gender, demographic features, clinical features, and treatment. ^a^Treatment within four weeks of coronavirus disease 2019 diagnosis.

Variables	N	Deceased	Surviving	P-value
Total patients	1,519	72 (4.74%)	1,447(95.26%)	
Age of the patients (median and min–max)	62 (20–95)	68 (43–86)	62 (20–95)	<0.001>
Gender				
Female	769 (50.6%)	19 (2.5%)	753 (97.5%)	
Male	750 (49.37%)	53 (7.06%)	694 (92.3%)	<0.001>
Age groups				
≤35	73 (4.80%)	1 (1.38%)	71 (97.26%)	<0.001>
36–50	274 (18.03%)	5 (1.82%)	269 (98.17%)	
51–65	589 (38.8%)	23 (3.90%)	565 (95.92%)	
>65	583 (38.39%)	42 (7.20%)	541 (92.79%)	
Hospital type				
Private	279 (18.3%)	18 (6.5%)	261 (93.5%)	
Public	1,058 (69.5%)	41 (3.9%)	1,017 (96.1%)	0.002
University	179 (11.78%)	18 (9.7%)	161 (89.94%)	
Comorbidities				
Diabetes	194 (12.7%)	19 (9.8%)	175 (90.2%)	0.004
Hypertension	391 (25.7%)	28 (7.2%)	363 (92.8%)	0.032
Chronic kidney disease	71 (4.73%)	6 (8.33%)	64 (88.9%)	0.025
Chronic obstructive pulmonary disease	104 (6.84%)	10 (9.61%)	94 (88.7%)	0.009
Chronic liver disease	31 (2.04%)	7 (22.58%)	24 (77.41%)	<0.001>
Cerebrovascular event	69 (4.54%)	4 (5.8%)	65 (94.2%)	0.77
Number of comorbidities				
0	530 (34.89%)	19 (3.58%)	511 (96.8%)	<0.001>
1	394 (25.93%)	17 (4.31%)	377 (95.6%)	
2	295 (19.42%)	6 (2.0%)	289 (98.0%)	
≥3	300 (19.74%)	30 (10%)	270 (90%)	
Cancer type				
Lung	159 (10.46%)	7 (4.40%)	152 (95.59%)	<0.001>
Colorectal	155 (10.20%)	17 (10.96%)	138 (89.03%)	
Breast	42 (2.76%)	3 (7.14%)	39 (92.85%)	
Skin	312 (20.53%)	6 (19.23%)	306 (98.07%)	
Renal	165 (10.86%)	9 (5.45%)	156 (94.54%)	
Prostate	48 (3.15%)	3 (6.25%)	45 (93.75%)	
Other	130 (8.55%)	10 (7.69%)	120 (92.31%)	
Thyroid and endocrine glands	144 (9.47%)	4 (2.77%)	140 (97.3%)	
Bladder	364 (23.96%)	34 (9.34%)	330 (90.65%)	
Targeted treatment^a^	55 (3.62%)	5 (9.1%)	50 (90.9%)	0.19
Cytotoxic treatment^a^	205 (13.5%)	20 (9.8%)	185 (90.2%)	0.003
Hormonal treatment^a^	169 (11.1%)	6 (3.6%)	163 (96.4%)	0.46

In total, 58% of patients underwent CT scans at least once. In 95.1% of patients, CT scan findings of the thorax were consistent with viral pneumonia. In the first PCR test, 87.6% of the patients presented with a confirmed COVID-19 diagnosis. The proportion increased to 97% in the second PCR test, and in the third test, the proportion reached 99.3%. Patients who presented with a negative PCR test in the first attempt showed a higher mortality rate compared with the positive ones (12.2% vs. 4.0%; p < 0.0001). Table 2 shows the laboratory results of positive cancer patients diagnosed with COVID-19.

**Table 2:** Analysis of laboratory results. Hb: hemoglobin

Variables	N	Values	Deceased	Surviving	P-value
Leukocyte (×10^3^/μL)	1,042	5.60 (4.2–7.5)	7 (4.9–13.4)	5.6 (4.3–7.5)	0.001
Hb (g/dL)	1,059	12.6 ± 1.99	10.9 ± 2.5	12.8 ± 2.01	<0.001>
Neutrophil (×10^3^/μL)	842	3.7 (2.7–5.6)	6.4 (4.1–13.3)	3.6 (2.6–5.2)	<0.001>
Lymphocyte (×10^3^/μL)	1,030	1.2 (0.8–1.7)	0.8 (0.4–1.3)	1.2 (0.8–1.7)	<0.001>
Neutrophil–lymphocyte ratio >4	831	4 (1.9–5.02)	8 (4.5–14.92)	3.01 (1.9–4.3)	<0.001>
Platelet (×10^3^/μL)	1,048	192 (152–247)	172 (108–258)	193 (154–247)	0.073
Fibrinogen (mg/dL)	209	439 ± 151	481 ± 249	433 ± 134	0.38
C-reactive protein (mg/L)	989	23.01 (7.7–75.8)	110 (60.1–200)	21.02 (7.4–64.3)	<0.001>
D-dimer (μg/L)	536	710 (385–1,329)	1,952 (1,048–4,989)	654 (380–1,095)	<0.001>
Ferritin (ng/L)	390	188 (80.2–405)	761 (155–1,310)	178 (79.1–390)	<0.001>
Procalcitonin (ng/mL)	340	0.2 (0–0.3)	0.3 (0.2–1.1)	0 (1–1.2)	<0.001>
International normalized ratio	439	2 (1.8–2.0)	1.0 (1.10–1.80)	1 (1.5–1.8)	0.001
Troponin-I (mg/L)	231	3.5 (0–8)	11.4 (5.2–31.1)	2.5 (0–5.2)	<0.001>
Lactate dehydrogenase (U/L)	639	242 (189–325)	385 (245–652)	242 (132–318)	<0.001>
Bilirubin (mg/dL)	592	0.5 (0.4–0.8)	0.7 (0.5–1.2)	0.3 (0.2–0.5)	0.001
Albumin (g/dL)	500	3.10 ± 0.4	3.1 ± 0.4	3.8 ± 0.6	<0.001>
Alanine aminotransferase (U/L)	859	22 (15–30)	21 (11–34)	22 (13–29)	0.68
Aspartate aminotransferase (U/L)	910	27.01 (20–36.5)	35 (21–63)	24 (20–34)	0.005
Alkaline phosphatase (U/L)	451	80 (60–110)	97.1 (81.1–150.1)	77 (59–100)	<0.001>
Gamma-glutamyl transferase (U/L)	480	31 (20.18–55.4)	57 (33–10.1)	31 (20–58)	<0.001>
Sodium (mEq/L)	510	137 ± 6	134 ± 8	139 ± 2	0.80
Creatinine (mg/dL)	851	0.9 (0.8–1.2)	0.8 (0.6–1.4)	0.6 (0.5–0.9)	0.036
Creatine phosphokinase (U/L)	445	73 (46–120)	69.10 (34–118.4)	74 (48–120)	0.74

In 73% of patients, specified COVID-19 therapy was performed, and the most common treatments were HCQ plus azithromycin in 45.16% and total HCQ in 69.7% of patients. Total favipiravir was given to 20.4% of patients to salvage refractory cases. This treatment was also used as first-line therapy for severe pneumonia patients and ICU patients. Since the outbreak of COVID-19 occurred during the influenza season, oseltamivir was employed in 51% of patients. The median follow-up time for our cohort was 15 days, with a minimum of one and a maximum of 74 days. In the study, 56.6% of patients were hospitalized and 7.1% of them died within 30 days. Among hospitalized patients, 11.32% were admitted to the ICU and 30.8% of them died; invasive mechanical ventilation was given to 7.4% of patients and 39.8% of them died. This analysis of treatment regimens and patient outcomes is shown in Table 3.

**Table 3:** COVID-19 treatment regimens and patient outcomes. HCQ: hydroxychloroquine; ICU: intensive care unit; COVID-19: coronavirus disease 2019

Treatment regimens	N	Deceased	Surviving	P-value
Total azithromycin	701 (45.8%)	39 (5.6%)	662 (94.4%)	0.34
Total HCQ	1,066 (69.7%)	59 (5.5%)	1,007 (94.5%)	0.25
Total lopinavir	46 (3.0%)	4 (8.7%)	42 (91.3%)	0.28
Total favipiravir	311 (20.4%)	47 (15.1%)	264 (84.9%)	<0.001>
Azithromycin + HCQ + favipiravir	218 (14.35%)	28 (16.27%)	190 (87.15%)	<0.001>
HCQ + azithromycin	687 (45.1%)	39 (5.7%)	648 (94.3%)	0.34
Supportive therapy alone	406 (26.7%)	10 (2.5%)	396 (97.5%)	0.005
Hospitalization	862 (56.6%)	61 (7.1%)	801 (92.9%)	<0.001>
ICU admission	172 (11.32%)	53 (30.8%)	119 (69.1%)	<0.001>
Intubation	113 (7.4%)	45 (39.8%)	68 (60.2%)	<0.001>

In a subset of data, we ran the multivariate model and included demographic and clinical information that was available at the time, including age, cancer type, diabetes, chemotherapy, and other comorbidities. The analysis is shown in Table 4.

**Table 4:** Univariate and multivariate regression analysis of potential baseline clinical variables. OR: odds ratio; COPD: chronic obstructive pulmonary disease; CKD: chronic kidney disease

Variables		Deceased	OR (univariable)	OR (multivariable)
Age groups	≤50 (ref)	3/347 (0.86)	—	—
	51–65	27/589 (4.6)	4.34 (1.49–12.45; p = 0.005)	3.61 (1.20–11.15; p = 0.018)
	>65	42/583 (7.2)	6.80 (2.35–18.60; p < .001)	5.20 (1.78–16.02; p = .002)
Gender	Male	54/750 (7.2)	3.31 (1.95–5.61; p < 0.001)	2.28 (1.24–4.19; p = 0.008)
	Female (ref)	18/769 (2.3)	—	—
Cancer site	Other* (ref)	21/339 (6.1)	—	—
	Colorectal cancer	9/164 (5.5)	0.90 (0.40–1.89; p = 0.761)	0.58 (0.25–1.30; p = 0.194)
	Gastric cancer	4/62 (6.4)	1.22 (0.44–3.40; p = 0.69)	0.79 (0.29–2.32; p = 0.680)
	Skin cancer	2/44 (4.5)	0.97 (0.29–3.40; p = 0.97)	0.80 (0.23–2.90; p = 0.748)
	Lung cancer	19/154 (12.3)	1.79 (0.94–3.39; p = 0.079)	0.97 (0.50–1.95; p = 0.95)
	Prostate cancer	7/165 (4.2)	0.69 (0.29–1.59; p = 0.391)	0.35 (0.14–0.90; p = 0.025)
	Breast cancer	1/301 (0.3)	0.08 (0.03–0.40; p = 0.001)	0.12 (0.02–0.61; p = 0.008)
	Thyroid cancer	2/139 (1.4)	0.11 (0.02–0.76; p = 0.025)	0.19 (0.03–1.34; p = 0.092)
	Bladder cancer	5/101 (4.9)	0.90 (0.35–2.20; p = 0.750)	0.49 (0.19–1.30; p = 0.140)
	Renal cancer	2/48 (4.2)	0.31 (0.04–2.38; p = 0.262)	0.26 (0.03–2.04; p = 0.200)
Diabetes mellitus	Yes	16/192 (8.33)	2.40 (1.41–4.10; p = 0.002)	2.15 (1.18–3.90; p = 0.006)
	No (ref)	56/1,327 (4.22)	—	—
COPD	Yes	10/104 (9.6)	2.70 (1.40–5.10; p = 0.003)	—
	No (ref)	62/1,415 (4.4)	—	—
CKD	Yes	5/70 (7.14)	2.49 (1.18–5.38; p = 0.021)	—
	No (ref)	67/1,449 (4.6)	—	—
Cytotoxic therapy	Yes	18/203 (8.9)	2.40 (1.35–4.10; p = 0.001)	2.12 (1.21–3.84; p = 0.009)
	No (ref)	54/1,316 (4.1)	—	—

## Discussion

Mortality in COVID-19 patients within the first 30-day period was found to be 4.74% in this study of adult patients with solid tumors. Lung cancer patients were found to be at a higher risk of mortality, whereas prostate cancer, thyroid cancer, and breast cancer patients showed lower mortality risk. Cytotoxic treatment, diabetes, old age, and time since cancer diagnosis were found to be crucial predictive factors of increased mortality risk. Lymphopenia was found to be a high mortality risk factor in laboratory outcomes, whereas the use of azithromycin and HCQ was found to have no effect on the 30-day mortality timeline. Compared with noncancer patients, the mortality rate of cancer patients was found to be higher in COVID-19 cases. The first report from China reported the case fatality rate (CFR) to be 28.6%. Two subsequent reports from China showed that the CFR was 20%. Solid tumor mortality of 12% and hematological malignancy of 14% were found in a study done by the COVID-19 and Cancer Consortium (CCC19). One study from the Memorial Sloan Kettering Cancer Center (MSKCC) reported the CFR as 12% [9-13].

ICU support was found to be high in Pakistan because the rate of patients who died from COVID-19 without admission to an ICU was lower. As the COVID-19 outbreak hit Pakistan later than it did China and Europe, ICUs were made available in higher numbers in Pakistan to comply with COVID-19 readiness. Seventy-one percent of patients who died underwent either invasive or noninvasive ventilation; this figure is found to be higher than those reported from the MSKCC, the CCC19, and the United Kingdom Coronavirus Cancer Monitoring Project series [13-15]. Higher availability of ventilation and intensive care can also be the reason for a lower mortality rate in Pakistan for COVID-19. As COVID-19 cancer patients were clustered nationwide, intensive filiation studies may also have contributed to lower COVID-19 severity in the study [16].

Cytotoxic treatment for weeks after the diagnosis of COVID-19 turned out to represent a high risk of mortality compared with endocrine therapy and targeted therapy. Some studies have contradicted these results in that they did not show a correlation between higher mortality rates and cytotoxic therapy. However, a study showed a positive correlation between recent cytotoxic therapy and the mortality rate in COVID-19 patients. It also presented a higher risk in patients who received cytotoxic treatment within two weeks, but if the time was more than three weeks, the risk was lower [17]. From the results of our study, we can conclude that during cytotoxic treatment, a pandemic patient who can move to alternative treatment should be shifted to safer therapies, and a patient who has no alternative treatment option should continue with cytotoxic treatment with the utmost care and strong protective measures against COVID-19. An unfavorable prognosis was presented in the MSKCC study for COVID-19 patients who received or were receiving immunotherapy [15]. In our study, we only had two patients who received immunotherapy, and this issue needs further analysis.

Nearly 50% of patients in our study group received azithromycin and HCQ treatment; this treatment is widely adopted and endorsed by the Ministry of Health in Pakistan for patients showing clear symptoms of COVID-19. A number of previous studies have shown that patients who receive HCQ treatment are at a high risk of mortality [18,19]. However, the results of our study differ from these studies because we could not find an increased risk of mortality in patients who were treated with HCQ with or without azithromycin compared with patients who did not receive these treatments. These results may be because patients underwent HCQ treatment as a consequence of higher risk factors and symptoms. As we used registry-based data in our study, it is safe to say that some of the risk factors were incomplete because of multiple assessments; hence, we did not compare our study with formal COVID-19 treatments.

A higher mortality rate is also correlated with higher international normalized ratio and D-dimer levels, suggesting coagulopathy. The poor prognosis of this disease is also correlated with gamma-glutamyl transferase, troponin I, alkaline phosphate, and creatinine. Consideration of these factors can be useful in the prognosis of early screening of critical disease, especially when determining inflammation dynamics and phases of immunity and early treatment [20,21]. However, these biomarkers are not available for each patient, so we did not use a single marker or a group of markers for our prognostic measures. Our study suggests that these biomarkers can be helpful in further studies.

The major limitation of our study was missing information on COVID-19 patients; we only included the PCR test, but the true number of COVID-19 patients may be higher. Moreover, a long-term follow-up of more than 30 days may compile better results on mortality in cancer patients as a result of COVID-19.

## Conclusions

COVID-19 has a higher prevalence in cancer patients than in the general population, resulting in higher mortality rates. In our study, we focused on solid tumor patients infected with COVID-19, and this is the largest series involving such patients to date. The results confirm previous reports’ clinical features and the higher mortality rate in this group of patients; however, the mortality rate was lower than that in China, as well as Western and European countries. Our study showed that clinical readiness and supportive intensive care can decrease mortality rates in COVID-19 and cancer patients, as well as patients with other life-threatening diseases. However, it is imperative to better identify treatment regimens and strategies from new interventional and observational studies of larger groups.
